# Reaction to and Coping With Domestic Violence by Iranian Women Victims: A Qualitative Approach

**DOI:** 10.5539/gjhs.v8n7p100

**Published:** 2015-11-17

**Authors:** Masoud Bahrami, Paymaneh Shokrollahi, Shahnaz Kohan, Ghodratollah Momeni, Mozhgan Rivaz

**Affiliations:** 1 Nursing and Midwifery Care Research Center, School of Nursing and Midwifery, Isfahan University of Medical Sciences, Isfahan, Iran; 2 Student Research Center, School of Nursing and Midwifery, Isfahan University of Medical Sciences, Isfahan, Iran; 3 Department of Nursing and Midwifery, Islamic Azad University-Firoozabad Branch, Iran; 4 Department of Islamic Study, Isfahan University of Medical Sciences, Isfahan, Iran; 5 School of Nursing and Midwifery, Shiraz University of Medical Sciences, Shiraz, Iran

**Keywords:** reaction, coping, domestic violence, qualitative approach, content analysis

## Abstract

**Introduction:**

Domestic violence is a continual stressor that motivates its victim to react. The way a woman deals with her husband’s violence determine the consequence of the violent relationship. In the present study, a qualitative approach was employed to investigate women’s reactions to and ways of coping with domestic violence.

**Method:**

Semi-structured interviews were conducted in 2014 with 18 women who experienced domestic violence in an attempt to explain how women deal with domestic violence. After the interviews were transcribed word by word, they were explored in the form of meaningful units and encoded as subcategories and categories through inductive content analysis. The reliability and validity of the interviews were measured by an external supervisor.

**Results:**

Two categories of reaction and coping were identified through content analysis: passive and non-normative measures and active measures. Passive and non-normative measures included the subcategories of harmful behaviors, retaliation, tolerance, and silence. Active measures included seeking help and advice, legal measures, leaving the spouse, positive and health promoting measures.

**Conclusion:**

In the present study, ways of coping with a husband’s violence among women experiencing domestic violence were divided into two categories: passive and non-normative measures and active measures. These categories confirmed the models of coping with stress in previous studies. Adopting an appropriate approach to dealing with domestic violence is affected by a woman’s capacity and beliefs, the dominant culture, intensity of the violence, available social and legal supports, and effectiveness of evaluation measures. To generalize service provision to victimized women, the type of coping and the reason for adopting the chosen approach need to be taken into account.

## 1. Introduction

For more than 2 decades, research in the fields of health, social problems, family, and jurisdiction has focused on domestic violence (Akyüz et al., 2008; Akyüz et al., 2012; Westbrook, 2008). A pivotal factor in the tendency toward studying domestic violence originates from the fact that this issue is considered as the basis for development at the intersection of several national and international movements in the fields of provision of health, improvement of health, and basic rights of women (De Jong, 2000; World Health Organization, 2000; World Health Organization, 2005). Moreover, since domestic violence can frequently occur in families in different forms, e.g., physical, emotional, spiritual, sexual, and economic violence, it can bring about more serious consequences compared with other types of violence against women (World Health Organization, 2013; Series, 2015). Over the centuries, the definition of domestic violence has changed from a normative phenomenon in families to a non-normative and harmful one that influences the family foundation (Anderson, 1997). Although young women are more likely to be affected by domestic violence, no social, economic, educational, ethnic, or racial class is secured against it (Heise & Garcia-Moreno, 2002). Most victims of domestic violence are women (Tjaden & Thoennes, 2006) who play the main role in childbirth and educating future generations; therefore, damages caused by domestic violence can impact several generations (Levendosky & Graham-Bermann, 2001; Sullivan et al., 2002)

Domestic violence is a continuous stressor that motivates the victim to react (Waldrop & Resick, 2004). The way a woman deals with her husband’s violence determines the consequence of the violent relationship. Coping refers to measures taken to reduce stress and its effects (Girdano et al., 2012). Studying and analyzing how women deal with domestic violence can lead to effective interventions to reduce or eliminate this phenomenon. Two strategies for coping with stressors have been explained in different studies: *approach* and *avoidance*, i.e. *behavioral* versus *cognitive* strategies.

The strategy of *approach* is adopting active behavior to reduce stress and its consequences. *Behavioral* strategy refers to measures that are observable and/or external. On the opposite side, *avoidance* and *cognitive* strategies refer to a lack of action or change in thinking in such a way that stressors can be avoided, such as denying the presence of violence or thinking a violent relation is the norm in order to better tolerate a violent life (Holahan & Moos, 1987; Tobin et al., 1989; Waldrop & Resick, 2004). In some cases, active coping, for instance quitting a violent relationship, might cause further threat to the victim instead of appropriate consequences (Rusbult & Martz, 1995). Sometimes female victims succumb to domestic violence either because of a lack of supportive resources or because the victims suppose violence to be normative. In some cultures, attempting to separate from a spouse instigates a negative cultural burden and leads to the loss of the advocacy of friends and acquaintances (Series, 2015; Yoshihama, 2002; Yoshihama et al., 2010). Lazarus and Folkman introduced another model of coping with stress (1984). In this model, two types of coping are introduced: *problem-focused coping*, which tries to adopt measures to remove violence, and *emotion-focused coping*, which seeks a negative emotional response (Lazarus & Folkman, 1984). The results of some studies have indicated that such models might not justify all measures (Parker & Lee, 2007), because several factors can influence the adoption of the coping type. For instance, one’s religion and the level of religiosity of individuals in a society can impact domestic violence and how it is dealt with (Baier, 2013). The results of a study conducted in Tehran on women who referred to forensic clinics revealed a correlation between the rate of self-efficacy, education, and subjective norms and the coping strategy and violence (Mohtashami et al., 2014). The method of coping with domestic violence can be understood in relation with one’s cultural and social background, the individual’s capacity, access to supportive resources, and the intensity of the violence (Waldrop & Resick, 2004). Finally, adopting an approach to cope with domestic violence depends on the victim’s evaluation of current conditions and how to survive under the stress (Dutton, 1996; Smith et al., 2010). Since domestic violence and coping with it are affected by cultural, social, and educational components, conducting the study in a certain regional context can be the most important success factor in better explaining and taking a step toward resolving domestic violence. That is why it can be claimed that quantitative studies alone cannot provide a complete understanding of this phenomenon. Therefore, conducting qualitative methods in studies on coping with violence among women in different cultures and social contexts can bring about appropriate results (Green, 2000). The present study was an attempt to explain coping with domestic violence in a specific cultural and social context by conducting content analyses on the interviews of 18 victims of violence.

## 2. Method

The present study was a qualitative research in which content analysis was employed. Its aim was to explain the coping experiences of purposefully-selected women in dealing with domestic violence. Inclusion criteria of the study were having experience with domestic violence, the ability to interact verbally with the researcher, and an informed tendency to express experiences. The study sample comprised 18 participants, some of whom had referred to health centers, others to forensic clinics, and some were selected through snowball sampling. After written or oral consent was obtained from the participants, they were interviewed by the researcher. While selecting the participants, the researcher attempted to include maximum variation. Interview locations were anywhere the participants felt comfortable, such as health centers or the researcher’s or participant’s home, place of study, or work space. Three participants were interviewed by telephone and the interviews were recorded, because the participants expressed a desire to remain anonymous. Sampling was continued until data saturation and lack of new data were obtained. After the 13th interview, no new data was observed; however, all 18 participants were interviewed to attain sureness. All interviews were conducted by the researcher (first author). The duration of the interviews varied between 20–50 minutes. All interviews were recorded and then transcribed. All transcribed texts and notes were considered by the researchers. Data was analyzed during data collection. The Graneheim and Lundman method was employed to conduct content analyses (Graneheim & Lundman, 2004). This method is an inductive approach in which the text is analyzed first in the form of meaning units, then by coding. By reviewing and categorizing similar codes and extracting the common meaning, the subcategories and categories were extracted. In order to ensure the validity of themes emerging from the interviews, this study’s two authors carefully documented the themes. Furthermore, the outside observer method was applied to evaluate similar perceptions with the investigator and search for inconsistent cases; by providing the initial codes that emerged from interviews and examples of how to extract the codes, and excerpts from interviews for each code were provided to an outside observer. During content analysis, the content was studied repeatedly, and the colleague’s opinions were asked as well. There was agreement over all themes. Other colleagues and authors were also asked for their views. Since the current study was part of a more extensive study aimed at designing a plan to enhance the health of women experiencing domestic violence, the interviews started with a general, open question, “What behavior does your husband have or did he have that hurt you?”, and continued with questions about the women’s feelings regarding violent behavior and adopting approaches to protect oneself according to the responses to violent behavior. Finally, the participants were allowed to express whatever they liked. In the first few interviews, the researcher decided how to direct semi-structured questions more purposefully.

Ethical considerations in this study included obtaining either written or oral consent from participants, ensuring them of confidentiality, and allowing them to freely participate in the research. Furthermore, sampling was done after permission was obtained from the Research and Ethics Committee of the School of Nursing and Midwifery, Isfahan University of Medical Sciences.

## 3. Results

The current study was conducted on 18 women from Isfahan and Shiraz who experienced domestic violence. A summary of the demographic information of female victims is presented in Table 1. Participants were aged 21–48 years. Education levels ranged from elementary levels to doctorate degrees. Two were divorced, 5 were separated and about to divorce, and the rest were continuing their married lives.

**Table 1:** Charactristic of domestic violence victims

characteristic	number	
Employment status	Employed	5
	housewife	8
	Student	5
Educational level	Under diploma	5
	Diploma	2
	University degree	11
Number of children	No child	6
	2-Jan	9
	More than 2	3
Marital status	Divorced	2
	Going to divorce	5
	Living together	11
Marriage duration	1-5 years	5
	5-10 years	4
	More than 10 years	9

Two main categories, non-normative and passive measures and active measures, were extracted as a result of content analyses of the interviews. Non-normative and passive measures included harmful behaviors, retaliation, tolerance, and silence, and active measures included seeking help and advice, legal measures, leaving the spouse, and positive and promoting measures; they are presented in Table 2.

**Table 2:** Methods for coping with domestic violence among victimized women

Codes	Subcategories	Categories
Suicide, tobacco consumption	Harmful behaviors	Passive and non-normative measures
Illegal affairs, ignoring the husband, sexual ignorance	Retaliation	
Accepting the violence and the situation, accepting the violation of an individual’s rights, accepting violence as normal, cultural belief in silence and tolerance	Tolerance and silence	
Referring to family consultants, psychologist, or legal consultant, talking to trustworthy individuals, attracting other’s support	Seeking help and advice	Active measures
Referring to legal entities to get a divorce and/or reduce the domestic violence	Taking legal measures and leaving the husband	
Pursing studies and gaining skills, adopting a better lifestyle like losing weight and exercising, seeking jobs and financing	Positive and promoting measures	

### 3.1 Non-Normative and Passive Measures

This category included measures that had no consequences other than harming the victim of violence. Emotional reactions, inability to cope with violence, lack of support, and frequency of violence resulted in a wrong orientation in dealing with the violence. Subcategories of this category included harmful behaviors, retaliation, tolerance, and silence.

### 3.1.1 Harmful Behaviors

While encountering a husband’s violence, measures related to threats to the individual’s health that could have been involved with early or late damages included suicide attempts or threats. Suicide attempts originated from despair and depression over continuing the violent life or attracting the husband’s attention or support. Participant #3, 40 years old, who experienced her husband’s physical and emotional violence said, “I’m frustrated. I even took a handful of drugs a while ago to kill myself, but unfortunately I failed. Only my liver was damaged.” Participant #2, 28 years old, who was experiencing her husband’s emotional violence and negligence stated, “I’ve been so severely stressed by the atmosphere at home that I’d like to commit suicide; I can even take 10 to 20 pills.” Some participants referred to beginning tobacco consumption as a response to violent measures of their husband. “Sometimes I’m so tired of my husband’s behavior that I resort to smoking cigarettes or hookah,” said Participant #2, 28 years old.

### 3.1.2 Retaliation

Retaliatory measures were another adopted non-normative measure for dealing with a husband’s violence. Establishing a sexual relationship with a stranger to compensate for a lack of affection in the marital life was another measure that potentially exposed the individual to social, emotional, and physical damages by sexually transmitted diseases. “I’ve had affairs with 15 men so far. My husband’s apathy is the reason. Last night he cursed me a lot,” said Participant #3, 21 years old. She continued, “Others love me; that is why I have affairs with them.”

Since the sexual behavior of women compared with that of men is formed with mental and emotional motivation, sexual ignorance was named by the participants as a retaliatory behavior against a husband’s violence or an unconscious reaction. Because of feelings of humiliation, oppression, and mistrust of their husbands, female victims are reluctant to have a sexual relationship or they use sexual ignorance as a means of retaliating against their husbands’ violence. Participant #7, 48 years old, reacting to her husband’s controlling behaviors, stating, “It does no good if I pay sexual attention to him; his behavior won’t change. I’m sick of him. Above all, he expects me to caress him.”

### 3.1.3 Tolerance and Silence

One behavior for coping with a husband’s violence noted in the present study was tolerance and silence in order to maintain peace in the family and for the children. The participants declared that by practicing passive treatment, they consciously decide to prevent their children from experiencing damage from the domestic violence.

Participant #6, 46 years old, commented on coping with her husband’s behavior of not recognizing women’s right to use television or electricity at their will stating, “He tells me not to turn up the light, so I turn it off or leave the room. Just for the sake of the kids and to keep peace in the family, I forget all about it and do not argue with him.”

“I decided not to talk to him, because it was quite in vain. Let him say whatever he likes; I nod at him pretentiously just to prevent quarrels and tension in the house. Just for the sake of the kids, I didn’t want to continue the argument and quarrel,” said Participant #5, 45 years old. She continued, “I’m not important at all. I just want to do something to prevent my husband from making quarrels and arguments so my kids can be safe.”

## 3.2 Active Measures

In the present study, active measures referred to behaviors that, although they might not be effective in reducing violence or removing the victim from the violent situation, at least the victim had a correct orientation, and, in some cases, these measures resulted in empowering female victims and changing their beliefs, knowledge, and awareness.

### 3.2.1 Seeking Help and Advice

This category refers to the individual’s attempt to seek help, awareness, and knowledge to improve their relationship or reduce the mental drain. This measure was carried out by referring to family counseling or legal centers or by talking about the domestic violence with family members and friends to gain their support. In some cases, this measure had no obvious results. One reason for this is the lack of the husband’s interaction with his wife or the thought that the domestic violence was deeply rooted.

“A while ago I referred to a counseling center. They told me to begin from myself and to try to take my husband with me, too. My only problem is that I don’t know how to take him to the center.” Said participant #8, 30 years old

Participant #7, 48 years old, responding to her husband’s paranoid behavior, and said, “I’ve even told him that he is sick and he should seek treatment. We referred to a psychologist and a psychiatrist, but no results were gained. He told me that I got him sleeping pills so he would fall asleep and I could go hang out.” In some cases, seeking help and advice led to thinking and understanding the situations or in enabling the individuals through gathering awareness and knowledge.

“I went to a consultant. I don’t know; I didn’t feel I could do anything that time. He told me that I deserved more than this life. This left a great influence on me. I came to my senses and tried to change my life and value myself,” said Participant #5, 45 years old.

In some cases, talking with another individual was mentioned as a major need of female victims of domestic abuse for mental drain. “I just need someone to listen to me,” reported Participant #11, 24 years old.

### 3.2.2 Legal Measures and Leaving the Spouse

While the first measure adopted by most women against their husband’s violence was silence and tolerance, some attempted to leave their marriage temporarily and/or divorce when they got exhausted and frustrated of improving their relationship. “I think it’s impossible. A tooth that aches should be pulled out and thrown away when you cannot restore it. You need to separate from him so you can reach spiritual peace and calmness. I also want to get separated,” said Participant #3, 47 years old.

By taking their concerns to legal centers, some women tried to stop their husbands’ violence; however, most of them failed. Participant #3, experiencing her husband’s violence, said, “Once after I was hit by him, I went to the court and lodged a complaint, but it led to nothing. They took him to the police station, and then his family bailed him out. The only result was that he became more violent against me.”

Some violence victims continue married life because they lack social support, family support, or financial resources, or because they believe legal measures are futile, or because of cultural issues, like viewing divorce as a shame or believing in biting the bullet.

### 3.2.3 Positive and Promoting Measures

Some of the participants of the current study referred to their husbands’ violence as an opportunity to adopt positive measures. Focusing on positive behaviors, they tried to reduce the pressure of domestic violence. Positive behaviors included education, employment, and further attention to self-care behaviors, like paying attention to physical health, playing sports, paying more attention to diet, losing weight, paying more attention to appearance, and trying to reduce tobacco consumption. “My main reason for attending university was to be away from home. While I’m at university, I’m free from thoughts of how my husband acts toward me,” said Participant #11, 25 years old.

Although pursuing positive behaviors was not aimed at reducing violence, according to the participants, it did result in improving their lives. After behaving positively, the victimized women felt a sense of empowerment which led to further hope for the future. Participant #11 said, “The reason that I tolerate my husband is that I think about life after him. I feel I can manage my life then.”

## 4. Discussion

In the present study, content analysis was carried out on measures taken by female victims of domestic violence to deal with their husbands’ violence. Content analysis led to the recognition of passive and non-normative behaviors that included the subcategories of harmful behaviors, retaliation, tolerance, and silence, which are classified as cognitive coping and/or avoidance or, according to Lazarus and Folkman, emotion-centered copings. The other category recognized was active measures, which included subcategories of seeking help and advice, legal measures, leaving the spouse, and positive and promoting measures which can be classified as approach or behavioral coping and/or problem-based coping (Graneheim & Lundman, 2004; Lazarus & Folkman, 1984; Tobin et al., 1989; Waldrop & Resick, 2004). The category of non-normative and passive measures included harmful behaviors like suicide and tobacco consumption, retaliation, and silence. The origin of this type of coping can be found in the inability and lack of necessary knowledge about supportive resources and mental damage incurred during a violence-filled life. Such a life can cause stress and tension in the victims, and that can lead to mental disorders and non-normative reactions (Black, 2011; Campbell & Lewandowski, 1997; Graneheim & Lundman, 2004; Lazarus & Folkman, 1984; Tobin et al., 1989; Waldrop & Resick, 2004). The results of a meta-analysis conducted by Golden et al. (1999) indicated that there is a relationship between a husband’s violence and such disorders as depression, attempted suicide, and addiction to alcohol and narcotics (Golding, 1999). The results of a study conducted in a poisoning emergency center indicated that women’s attempts at suicide are due to marital problems and their husbands’ violence (Memari et al., 2006).

Another subcategory of passive measures was silence and tolerance toward a husband’s violence. Silence is more common coping strategy to husband’s violence (Nasrabadi et al., 2014). Although the women considered violence serious, they chose a high level of tolerance toward the violence and the denial of it, which can have originated in a desire to avoid tension in the family, protect the children against violence, and/or receiving blame from the dominant culture. In some cultures, active measures against domestic violence, like leaving an abusive husband, have negative cultural burdens (Kasturirangan et al., 2004; Sadeghifasai, 2010; Yoshihama, 2002). In the present study, some of the women adopted the strategy of silence in order to maintain their children’s peace and because they believed that any reaction to violence would be futile. Silence was classified in this study as a passive measure because of its ineffectiveness in changing the conditions of domestic violence. In Goodness-of-Fit Theories of coping, however, selecting passive measures like tolerance is believed to be due to the victimized individual’s evaluation of the uncontrollable situations of life, which in some cases seem to save an individual’s security (Yoshihama, 2002). There are two views in coping with stress. One is to respond to stress from an inter-individual perspective, which means to utilize one method in responding to stress., The other view is from an intra-individual perspective, which indicates that individuals change their coping approach according to their type of stress, i.e. although individuals prefer a certain strategy for responding to stressors, their response can change depending on the type of the stress and the situation in which they are; therefore, the conditions and type of stress should be considered when wanting to understand the coping completely (Waldrop & Resick, 2004). Studies indicated that changing one’s behavior, avoiding violence, and restricting one’s relationship with her husband are measures adopted by women who take passive approaches (Zink et al., 2006).

Another coping method was seeking help and advice. This measure has been known as an active approach in most studies (Bybee & Sullivan, 2002; Barrett & Pierre, 2011). The effectiveness or ineffectiveness of attracting support and help depends on the husband’s participation, the intensity or reason for the domestic violence, and also the individual’s empowerment, belief in changing the conditions, and the effectiveness of services provided by a center or individuals. Most women’s measures are usually aimed at attracting support from unofficial resources, like friends and family members, maybe because of the restriction or unavailability of special organizations for that purpose. On the other hand, if the intensity and complications of violence are severe, prolonged, and/or irresolvable, attempts to attract support take place more officially or unofficially (Macy et al., 2005; West et al., 1998).

Leaving the relationship and taking legal measures against a husband’s violence can occur in a violent relationship. Adopting this measure depends on the intensity of the violence, access to legal support, and the rate of dependence or independence in economic, emotional, and social regards. Avoiding leaving the husband and adopting legal measures can be due to the belief that such measures are futile in the legal and social context (Lewis et al., 2000; Waldrop & Resick, 2004) or because of environmental pressures and labels that entail such measures (Kasturirangan et al., 2004; Tonsing, 2015). Sometimes the victim of violence thinks that living in a violent house is better than having no place to live, especially in situations where leaving the children or leaving along with them would be a far more complex issue when there are no social and family supports. Leaving the relationship and taking legal measures can be effective when the individual is well prepared in cognitive terms, like believing in the abnormality of violence, being aware of supportive resources, emotional conditions, and finally in regard with behavior like having a proper plan for separation and life after separation (Hutchison, 2003; Westbrook, 2008).

Behaviors like pursuing studies, earning money, exercising, and focusing more on health and appearance were also classified as subcategories of positive coping and empowerment gain. Such behaviors result in remarkable changes in adopting and changing the type of coping with or even reducing violence by creating internal satisfaction, empowerment, and hope and enhancing the quality of life (Bybee & Sullivan, 2002; Fredrickson, 2001; Abedi et al, 2014). Women who were successful in becoming empowered will usually not be faced with violence and thus can cope with it in most cases. Most women victimized by violence are those who have to tolerate the situation due to a lack of empowerment, and those for whom the violent behavior will be intensified because the victim does not give an appropriate response. Providing comprehensive civil and social supports (financial, security, and legal) seems to be one of the methods to resolving violence and preventing violent behavior (Harvey et al., 2007).

## 5. Conclusion

In the present study, reacting to and coping with a husband’s violence fell under two categories: passive and non-normative measures and active measures. These categories support the models of coping with stress in previous studies. Passive and non-normative measures included the subcategories of harmful behaviors, retaliation, tolerance, and silence. Active measures included measures of seeking help and advice, legal measures, leaving the spouse, positive and health-promoting measures. Adopting an appropriate approach for dealing with domestic violence is affected by women’s empowerment and beliefs, the dominant culture, intensity of the violence, availability of social and legal supports, and effectiveness of evaluation measures. In order to generalize service provision to victimized women, the type of coping and the reason for adopting such approach need to be taken into account.

## 6. Study Limitations

Due to the limited number of participants who were purposefully selected and because each of the participants had her own special cultural, educational, and social characteristics, the results and findings of the present study cannot be generalized to other populations or samples.
